# Error Analysis and Compensation of a Laser Measurement System for Simultaneously Measuring Five-Degree-of-Freedom Error Motions of Linear Stages

**DOI:** 10.3390/s19183833

**Published:** 2019-09-05

**Authors:** Yindi Cai, Qi Sang, Zhi-Feng Lou, Kuang-Chao Fan

**Affiliations:** Key Laboratory for Micro/Nano Technology and System of Liaoning Province, Dalian University of Technology, Dalian 116024, China

**Keywords:** laser diode, multi-degree-of-freedom measurement, error motions, linear stage

## Abstract

A robust laser measurement system (LMS), consisting of a sensor head and a detecting part, for simultaneously measuring five-degree-of-freedom (five-DOF) error motions of linear stages, is proposed and characterized. For the purpose of long-travel measurement, all possible error sources that would affect the measurement accuracy are considered. This LMS not only integrates the merits of error compensations for the laser beam drift, beam spot variation, detector sensitivity variation, and non-parallelism of dual-beam that have been resolved by the author’s group before, but also eliminates the crosstalk errors among five-DOF error motions in this study. The feasibility and effectiveness of the designed LMS and modified measurement model are experimentally verified using a laboratory-built prototype. The experimental results show that the designed LSM has the capability of simultaneously measuring the five-DOF error motions of a linear stage up to one-meter travel with a linear error accuracy in sub-micrometer and an angular error accuracy in sub-arcsecond after compensation.

## 1. Introduction

Linear stages, which are expected to travel along a straight line and precisely stop at some prescribed positions, play a crucial role in the manufacturing field and measurement field [1,2,3,4]. However, due to the manufacturing imperfections and the assembling errors, the linear stage inherits six-degree-of-freedom (six-DOF) error motions, including three linear errors (positioning error, horizontal and vertical straightness errors) and three angular errors (pitch, yaw and roll errors). These geometric errors of the linear stage will seriously affect the accuracy of the manufactured parts process or measured results. Compensation of these error motions is an effective approach to achieve a high accuracy [1,3]. To implement the error compensation, the six-DOF error motions should be accurately detected.

A laser interferometer, with high measurement accuracy and a long measurement range, is the most popular measurement instrument to identify these error motions [5,6]. However, the laser interferometer can only measure a single error with a specified optical accessory each time, which is extraordinary time-consuming to complete all errors. A laser tracker, which can simultaneously measure multi-DOF error motions, is another instrument usually applied to measure the error motions [7,8]. However, its measurement accuracy is low. In addition, the high cost is another drawback of the laser interferometers and the laser trackers.

A variety of systems, which are capable of simultaneously measuring the multi-DOF error motions, have been developed [1,2,9,10,11,12,13,14,15,16,17,18,19,20]. Measurement systems based on interferometry, such as the grating diffraction interferometry [9,10] and the laser synesthetic wavelength interferometry [2,11], have achieved a linear displacement resolution better than sub-nanometer and an angular displacement resolution of approximately sub-arcsecond. However, the measurement systems always have a complex configuration and time-consuming alignment process. The measurement accuracy of those systems is dependent on the characteristics of the laser source. In comparison, the non-interferometry-based measurement systems have been verified to be superior to the interferometry-based measurement systems in many aspects, such as simple construction and large measurement range [1,12,13,14,15,16,17,18,19,20]. Therefore, they are widely used for measuring the error motions of precision machines, such as numerically controlled machine tool and coordinate measuring machine.

The laser source of the above-mentioned systems could be the He-Ne laser [2,10,11,12], fiber laser [14,15,19,20], laser diode [9,17], or commercial laser interferometer [13,16,18]. However, no matter what kind of laser is adopted, there always exists the physical phenomenon of angular drift of the laser beam, which will influence the system’s measurement accuracy and repeatability. The common-path method is a feasible approach for compensating the errors induced by the laser beam drift in laser measurement system [14,21]. However, the common-path method makes the system complex and time-consuming for laser beam alignment. Different turning mirror mechanisms have also been proposed to actively compensate for this angular drift [17,22]. In our previous research on a four-DOF measurement system [17], an active compensation method was developed to reduce the effect of the beam angular drift of the laser diode on the measurement accuracy. However, the four-DOF measurement system cannot measure the roll error of the linear stage and the measurement errors of the system, which significantly influence the system measurement accuracy, were not analyzed.

Additionally, photodetectors are widely selected as the error detectors in the above-mentioned laser measurement systems [12,13,14,15,16,17,18,19,20]. The photodetector sensitivity is influenced by the laser diameter and intensity, which have relationships with the measurement distance along the laser propagation direction [23]. Therefore, it is essential to compensate for the errors caused by the sensitivity variation of a photodetector, especially in the long-distance measurement. However, the compensation of photodetector sensitivity was not considered in the above-mentioned measurement system [9,10,11,12,13,14,15,16,17,18,19,20]. In our previous research, a mathematical model was proposed for compensating the sensitivity variation of photodetectors, which were used as straightness error detectors [24]. However, it has not been verified whether this model can also compensate for the sensitivity variation of angular errors detectors.

Furthermore, since the stage is already equipped with a position sensor, either a linear scale or a rotary encoder, the stage’s positioning error can be easily obtained by the calibration with a commercial laser interferometer. Hence, from our point of view, a five-DOF error measuring system is adequate for each linear stage. Therefore, in this paper, a compact and low-cost laser measurement system (LMS) based on the principle of the laser collimation is proposed to simultaneously measure two straightness errors and three angular errors of linear stages, whose travel distance is larger than one meter. As mentioned above, there are some errors that would influence the accuracy of the LMS, including the laser beam drift, the photodetector sensitivity variation, dual-beam non-parallelism, and crosstalk errors. In addition to the integration of error compensation schemes that have been considered in our previous four-DOF [17] and roll measurement systems [24], the elimination of overall crosstalk errors is further considered and analyzed in detail in this report. This five-DOF LMS is designed in modular type, including the sensor head and the detecting part. Differ from the above-mentioned multi-DOF measurement systems that the sensor head and the detecting part are mounted on the base of the stage at the same side [14,19,20,21], the detecting part of this LMS is directly mounted on the moving stage so as to reduce the laser path distance induced errors. Additionally, a wireless transmission kit is applied in the electronic unit of the system, making the LMS is suitable for on-machine measurement. Based on the analyzed results, the compensation methods are proposed, and the measurement model is modified, which makes the LMS robust and precise.

## 2. Measurement Principle

### 2.1. Configuration of the LMS

**Figure 1:**
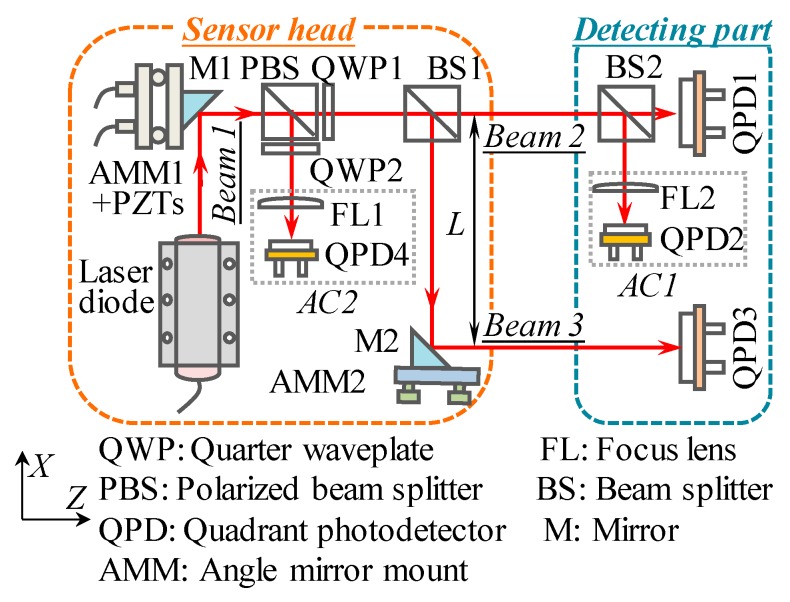
A schematic of the optical configuration for the five-degree-of-freedom (five-DOF) laser measurement system.

A laser measurement system (LMS) was designed to simultaneously measure the two straightness errors (*δ_x_* and *δ_y_*) and three angular errors (*θ_x_*, *θ_y_* and *θ_z_*) of linear stages. Figure 1 shows the schematic of the optical configuration for the LMS, which is composed of a sensor head and a detecting part.

A laser beam is divided into two beams by a polarizing beam splitter (PBS). The reflected beam (beam 1) enters into an autocollimator set (AC2) to detect the drifted angular errors (α_x_ and α_y_) of the laser beam. The transmitted beam is further split into two beams by a beam splitter (BS1), namely, beam 2 and beam 3. Beam 2 is projected onto two quadrant-photodetectors (QPD1 and QPD2) of the detecting part to measure *δ_x_*, *δ_y_*, *θ_x,_* and *θ_y_*. Beam 3 is projected onto QPD3 after bent by a mirror (M2) to measure *θ_z_*. It should be noted that the reflected beams from optical components are prevented from go back to the laser diode by using a polarization module, which consists of a PBS and two-quarter waveplates (QWP1 and QWP2) in the sensor head. The sensor head is fixed on the base of the linear stage and kept stationary during the measurement process. The detecting part, including a BS2, a focus lense (FL2) and three QPDs, is mounted on the moving table of the linear stage to detect the position variations of beam spots on each QPD induced by the error motions of the stage. Prior to measuring the error motions of the linear stage, particular effort is put into aligning beam 2 with the moving axis of the linear stage. In this alignment, QPD1 is employed to detect the lateral motions of the moving stage with respect to beam 1 in the *X*- and *Y*-directions. A rotary stage under the sensor head is adopted to adjust the direction of beam 2 to make its spot always close to the center of the QPD1 during the motion of the moving stage. Beam 3 is, then, adjusted to be parallel with beam 2 assisted by a commercial electronic level. The electronic level is mounted on the top of the detecting part. AMM2 is applied to adjust the direction of beam 3 so that the roll error measured by the LMS is the same as by the electronic level. The principle of roll error measurement will be detailed in Section 2.4.

### 2.2. Measurement Principle of the Straightness Errors

**Figure 2:**
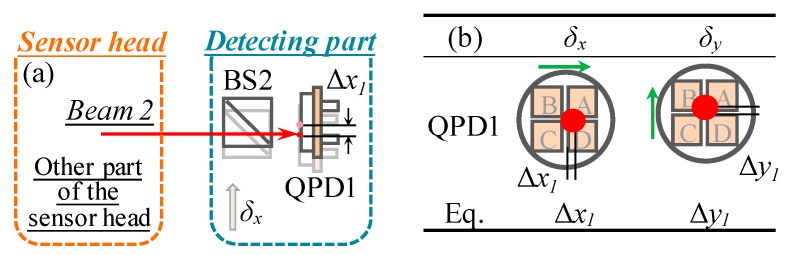
Measurement principle of the straightness errors: (**a**) Optical layout of straightness errors measurement kit, (**b**) variations of laser beam positions on quadrant-photodetectors (QPDs).

Laser beam 2, which is projected onto QPD1, can be treated as a reference straight line with respect to the linear stage after careful adjustment. Beam 2 will be located at the center of QPD1 when the linear stage has no straightness errors. Otherwise, the center of QPD1 will be shifted with respect to the laser beam, as shown in Figure 2a. The *X*- and *Y*-directional shifted distances of the QPD1 are recorded as Δ*x*_1_ and Δ*y*_1_, respectively, as shown in Figure 2b. Therefore, the straightness errors (*δ_x_* and *δ_y_*) can be obtained from the following equations:

(1) $$\delta_{x}=\Delta x_{1}=k_{x1}\cdot \Delta I_{x1}\;\mathrm{and}\;\delta_{y}=\Delta y_{1}=k_{y1}\cdot \Delta I_{y1}$$

where, *k_x*1*_* and *k_y*1*_* are the sensitivity of QPD1 in the *X*- and *Y*-directions, respectively. Δ*I_x*1*_* and Δ*I_y*1*_* indicate the output photocurrents corresponding to Δ*x*_1_ and Δ*y*_1_, respectively.

According to Equation (1), the sensitivity of QPD1 significantly influences the measurement accuracy of the straightness errors. It has been verified that the detector sensitivity varied with the measurement distance [21,24], since the diameter and intensity of the laser beam change along its propagation direction [23]. Therefore, the *X*- and *Y*-directional sensitivities *k_x_*_1_ and *k_y_*_1_ of QPDs should be corrected along the laser propagation direction. The *X*- and *Y*-directional sensitivities of QPD1 at the first measurement position *p*_0_ are set to be *k_x_*_1-0_ and *k_y_*_1-0_, respectively. *k_x_*_1*-f*_ and *k_y_*_1*-f*_ represent the *X*- and *Y*-directional sensitivities of QPD1 at the final measurement position *p_f_*, respectively. *k_i_*_1*-*0_ and *k_i*1*-f_* (*i* = *x*, *y*) can be calibrated by the comparison between the photocurrent of QPD and the output of a commercial displacement sensor. Thus, the following relationship can be obtained from a series of calibrated results by linear interpolation,

(2) $$k_{i1-d}=k_{i1-0}+(p_{0}+p_{d})\cdot \frac{k_{i1-f}+k_{i1-0}}{p_{f}-p_{0}}\;i=x,\;y$$

where, *k_i_*_1*-d*_ represents the sensitivity of QPD1 at the random measurement position *p_d_*, which is between *p*_0_ and *p_f_*. The sensitivity of QPD2 and QPD3 at *p_d_* also can be corrected by Equation (2). Without special description, the detector sensitivities used in the following descriptions and experiments are all corrected by Equation (2).

### 2.3. Measurement Principle of the Pitch and Yaw Errors

**Figure 3:**
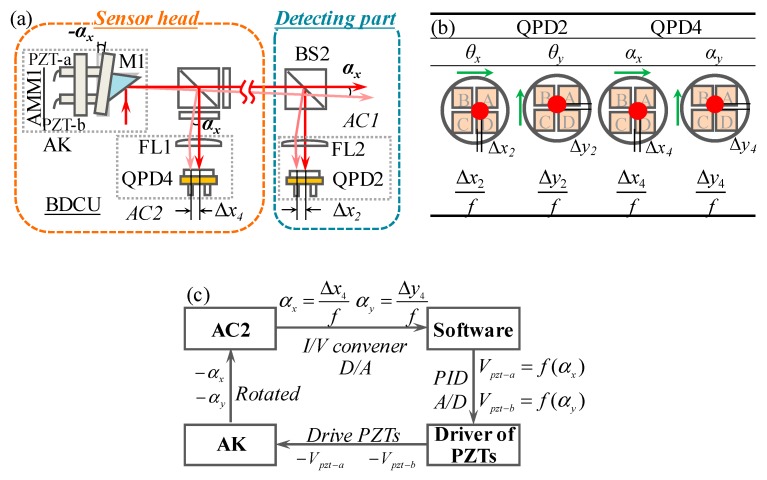
Measurement principle of the pitch and yaw angular errors: (**a**) Optical layout of pitch and yaw angular errors measurement kit, (**b**) variations of laser beam positions on QPDs, (**c**) procedure of compensating the drifted angular errors.

The pitch and yaw errors (*θ_x_* and *θ_y_*) can be measured based on the 2D autocollimation principle. Figure 3a shows a designed autocollimator set (AC1), which is composed of FL2 and QPD2. The distance between FL2 and QPD2 is *f*, which is the focal length of FL2. When the linear stage is rotated with an angle of *θ_x_* or *θ_y_*, the position of the laser-focused spot on QPD2 is moved along *X*-direction or *Y*-direction with Δ*x*_2_ or Δ*y*_2_, as shown in Figure 3b. Therefore, *θ_x_* and *θ_y_* can be calculated by

(3) $$\theta_{x}=\frac{\Delta x_{2}}{f}=\frac{k_{x2}\cdot \Delta I_{x2}}{f}\;\mathrm{and}\;\theta_{y}=\frac{\Delta y_{2}}{f}=\frac{k_{y2}\cdot \Delta I_{y2}}{f}.$$

It is known that within the laser beam exist drifted angular errors, which are induced by the thermal deformations of the laser diode cavity, the variation of the air refractive index and the fluctuation of the gas-films [22]. The laser beam drift would induce measurement errors of all QPDs. Therefore, a beam drift compensation unit (BDCU), consisting of an autocollimator (AC2) and an automatic angle adjustment kit (AK), is developed to compensate the drifted angle of the laser beam based on the proposed compensation principle in our previous research [17], as shown in Figure 3a. The AK is composed of an angle mirror mount (AMM1), two PZTs and a mirror (M1). The AC2 consists of FL2 and QPD2 is used to detect the drifted angular errors (*α_x_* and *α_y_*). The detections of *α_x_* and *α_y_* are based on the 2D autocollimation principle. *α_x_* and *α_y_* can be obtained from,

(4) $$\alpha_{x}=\frac{\Delta x_{4}}{f}=\frac{k_{x4}\cdot \Delta I_{x4}}{f}\;\mathrm{and}\;\alpha_{y}=\frac{\Delta y_{4}}{f}=\frac{k_{y4}\cdot \Delta I_{y4}}{f}$$

The procedure of compensating the drifted angular errors is shown in Figure 3c. A model, which represents the relationships between the beam drifted angular errors (*α_x_*, *α_y_*) and the driver voltage (*V_pzt-a_*, *V_pzt-b_*) of PZTs, is established. *V_pzt-a_* and *V_pzt-b_*, thus, can be evaluated based on the detected *α_x_* and *α_y_*. The angle of M1 is rotated to the opposite direction of *α_x_* and *α_y_* by two driven PZTs, from which the laser beam spot can remain in the center of the QPD4. The drifted angular errors, thus, can be automatically compensated. It should be noted that the BCDU is working during the experiments.

### 2.4. Measurement Principle of the Roll Error

**Figure 4:**
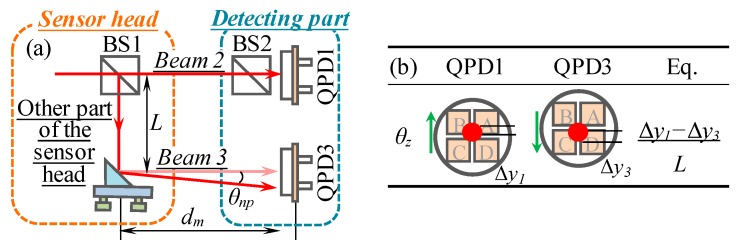
Measurement principle of the roll angular error: (**a**) Optical layout of roll angular error measurement kit, (**b**) variations of laser beam positions on QPDs.

Figure 4a shows the measurement principle of the roll error (*θ_z_*), which is carried out by detecting the relative position shift of two parallel laser beams (beam 2 and beam 3) in the *Y*-direction. Firstly, the laser beams are carefully aligned to the center of the QPD1 and QPD3. When the linear stage has a roll error, the *Y*-directional position of laser beams on QPDs will be varied, as shown in Figure 4b. According to the varied positions (Δ*y*_1_ and Δ*y*_3_), the roll error can be obtained from the following equation,

(5) $$\theta_{z}=\frac{\Delta y_{1}-\Delta y_{3}}{L}=\frac{1}{L}(k_{y1}\cdot \Delta I_{y1}-k_{y3}\cdot \Delta I_{y3})$$

where, *L* is the distance between beam 2 and beam 3 in the *Y*-direction.

As seen from Figure 4a, the non-parallelism of beam 2 and beam 3 significantly influences the position of two laser beams on QPDs. Therefore, the compensation of the non-parallelism induced by the misalignment and the laser beam drifted angular errors is essential for roll error measurement to achieve a high-precision measurement accuracy. If two laser beams are always parallel, *θ_z_* can be calculated by Equation (5). However, when two beams are non-parallel, an angle error *θ_np_* will exist in the output of LMS. According to geometric optics, *θ_z_* will be changed to be

(6) $$\theta \prime_{z}=\frac{\Delta y\prime_{1}-\Delta y\prime_{3}}{L}=\frac{\Delta y_{1}-\Delta y_{3}+p_{d}\tan\theta_{np}}{L}=\theta_{z}+\frac{p_{d}\theta_{np}}{L}$$

where, $\frac{p_{d}\theta_{np}}{L}$ represents the error component induced by the non-parallelism of beam 2 and beam 3. *θ_np_* is a constant and can be obtained by comparing measurement results using the designed LMS and a commercial electronic level. The outputs of the LMS (*θ_z-L_*) and electronic level (*θ_z-e_*) are reset to be zero at *p*_0_. Ideally, it is desired that the output of the two instruments should be the same at *p_f_*. However, due to the existence of *θ_np_*, the following equation can be obtained,

(7) $$\theta_{z-L}+\frac{p_{f}\theta_{np}}{L}=\theta_{z-e}$$

From Equation (7), *θ_np_* can be calculated. Substituting Equation (5) and Equation (7) into Equation (6), the error component induced by the non-parallel of two laser beams can be compensated.

## 3. Testing of the Designed LMS

A laboratory-built prototype of the LMS was constructed to measure the five-DOF error motions of a linear stage based on the described measurement principles and compensation principles.

A collimated laser beam emitted from a low-cost laser diode (DA635, Huanic, China) has a diameter of 5 mm and a divergence angle less than 0.2 mrad. The distance *L* between beam 2 and beam 3 was set to be 120 mm. Two high precision QPDs (QPD1 and QPD3, QP50-6-TO8, First Sensor, Germany) with an active area of 11.78 mm per element and a measurement resolution of 0.1 μm were chosen as the detectors of the straightness errors (*δ_x_* and *δ_y_*) and roll error (*θ_z_*). Therefore, the theoretical resolution for *δ_x_*, *δ_y,_* and *θ_z_* were evaluated to be 0.1 μm, 0.1 μm, and 0.17 arcsec, respectively. Two high precision QPDs (QPD2 and QPD4, QP5.8-6-TO5, First Sensor, Germany) with 4 × 1.44 mm^2^ active area and a measurement resolution of 0.05 μm were applied as the detectors of pitch and yaw errors (*θ_x_* and *θ_y_*) and the beam drifted angular errors (*α_x_* and *α_y_*). The focal length of FL used in ACs (AC1 and AC2) is 20 mm, the theoretical resolution for *θ_x_*, *θ_y_* and *α_x_, α_y_* are, thus, evaluated to be 0.25 arcsec. Two PZTs (AL1.65 × 1.65 × 5D-4F, NEC TOKIN Electronic, Sendai Janpan), an angle mirror mount (AMM1, GCM080205M, DHT Co., Beijing, China) and a right-angle mirror (M1, Foctek, Fujian, China) were used to construct the AK shown in Figure 3a.

**Figure 5:**
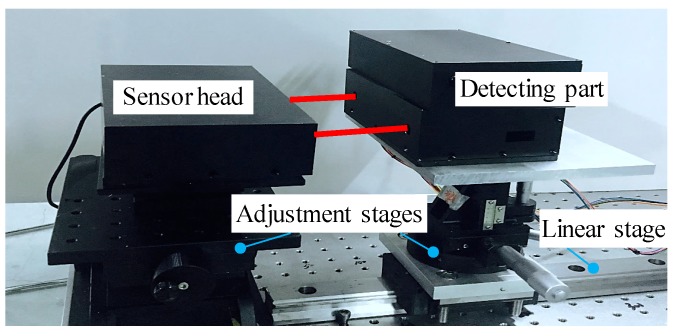
Experimental setup of the prototype laser measurement system (LMS).

Figure 5 shows the photo of the prototype LMS supported by adjustment stages which are mounted on a linear stage more than 1.2 m long. The LMS consists of a sensor head and a detecting part. Different from most of other five-DOF or six-DOF measurement systems that reflect the light from the moving part to the sensor head, this developed LMS directly mounts the detecting sensors for five-DOF error motions on the moving stage. The main reason is to reduce the laser path distance so as to reduce the errors caused by the laser beam. In this LMS, a wireless transmission kit is used in the electronic unit to avoid errors caused by the push and pull of the cable. Such a prototype LMS possesses innovative features of compact, portable, easy installation, wireless, and low-cost. It is suitable for on-machine measurement.

### 3.1. Stability of the LMS

**Figure 6:**
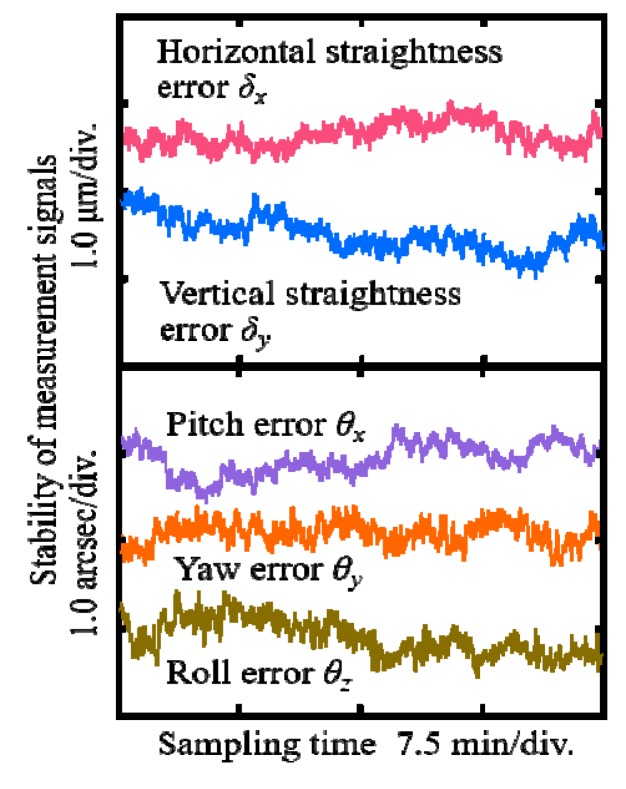
Stability of the measurement signals when the beam drift compensation unit (BDCU) was activated.

The stability of the LMS significantly influences the measurement accuracy of the error motions. The distance between the sensor head and the detecting part was first set to be 1.0 m for testing the stability. The sampling time and sampling frequency were set to be 30 min and 100 Hz, respectively. A moving average filter and a Butterworth filter were applied to reduce the noise levels of the measurement signals. Figure 6 shows the stability of the measurement signals for five-DOF error motions when the BDCU was worked. The beam drifted angular errors can be controlled within ±0.01 arcsec using the BDCU although the figures are omitted for the sake of clarity. The stability of the measurement signals for *δ_x_*, *δ_y_*, *θ_x_*, *θ_y,_* and *θ_z_* was evaluated to be 0.7 μm, 0.8 μm, 0.8 arcsec, 0.5 arcsec, and 0.8 arcsec, respectively, in a non-environmental controlled open laboratory. It was verified that the stability of the LMS was satisfied for measuring the error motions of the linear stage.

### 3.2. Calibration Tests of the LMS

The measurement accuracy of the designed LMS was calibrated by a commercial measurement instrument in advance. The first (*p*_0_) and final (*p_f_*) measurement positions were set to be 0 mm and 1.08 mm, respectively.

**Figure 7:**
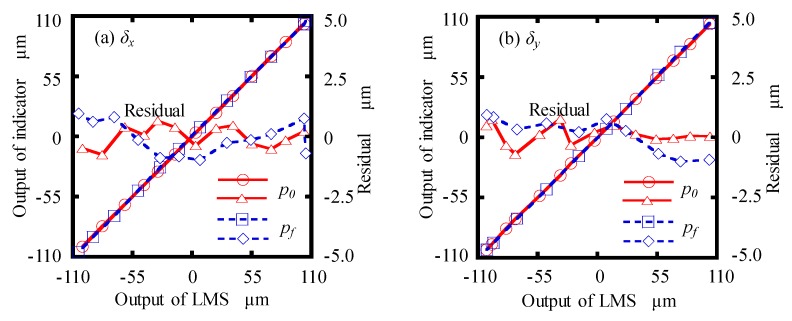
Calibration results of: (**a**) *δ_x_*; (**b**) *δ_y_.*

Firstly, using an *XY* manual linear stage, on which the detecting part was mounted, the motions were simultaneously detected by a commercial digital indicator (P12D HR, Sylvac, Switzerland), which has a resolution of 0.01 μm and a measurement accuracy of 0.22 μm. Since the commercial digital indicator could only detect one-directional displacement, the position of the digital indicator should be changed for the calibration along a different direction. The *X*- and *Y*-directional calibration results at *p*_0_ and *p_f_* are shown in Figure 7a,b, respectively. As seen in Figure 7a, the maximum residual of *δ_x_* were evaluated to ±0.7 μm and ±0.9 μm in the calibration range of ±100 μm at *p*_0_ and *p_f_*, respectively. Similar calibration results of *δ_y_* were also obtained, as shown in Figure 7b.

**Figure 8:**
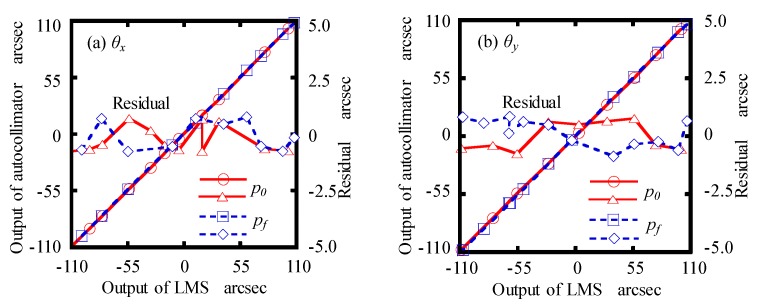
Calibration results of: (**a**) *θ_x_*; (**b**) *θ_y_*.

Then, a commercial autocollimator (5000U3050, AutoMat, China), with a measurement accuracy of 0.2 arcsec and repeatability of 0.05 arcsec, was used to calibrate the angular errors measurement performance of the LMS. The detecting part and the reflector of the autocollimator were mounted on a manual rotary stage and a manual goniometer stage. The angular displacements of the stages could be simultaneously measured by the LMS and autocollimator. Figure 8a shows the maximum residuals of *θ_x_*, which were found to be between ±0.6 arcsec and ±0.7 arcsec in the calibration range of ±100 arcsec at *p*_0_ and *p_f_*, respectively. The maximum residuals of *θ_y_*, which were estimated to be ±0.7 arcsec at *p*_0_ and ±0.8 arcsec at *p_f_* within the calibration range of ±100 arcsec, respectively, are shown in Figure 8b.

**Figure 9:**
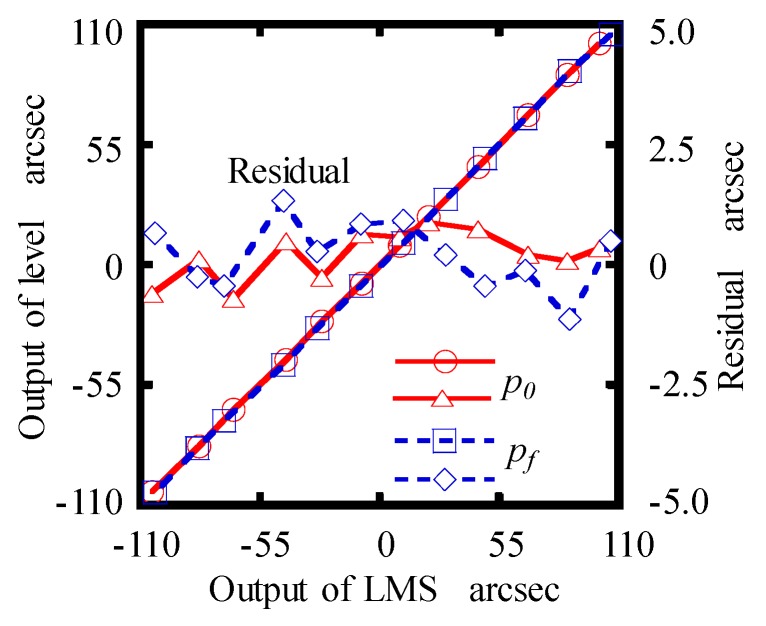
Calibration results of *θ_z_.*

Finally, a high precision commercial electronic level (WL/AL11, Qianshao, China), which has a measurement accuracy of 0.2 arcsec, was employed to calibrate the roll error measurement performance of the designed LMS. The detecting part and the electronic level were installed on a manual goniometer stage, which can be rotated about *Z*-axis. The maximum residual of each calibration, shown in Figure 9, was within ±0.7 arcsec and ±1.1 arcsec at *p*_0_ and *p_f_* in the calibration range of ± 100 arcsec.

Therefore, it was verified from the calibration results that the designed LMS can be acceptable for sub-micrometer precision straightness error measurement within ±100 μm and sub-arcsecond precision angular error measurement within ±100 arcsec.

### 3.3. Crosstalk Error Analysis of the LMS

**Figure 10:**
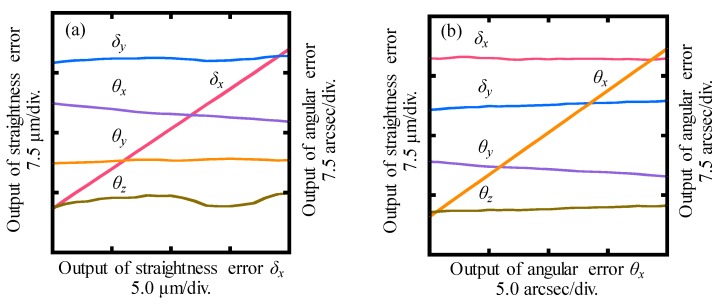
Crosstalk errors of the LMS when the sensor head was: (**a**) Moved along *X*-direction in the range of ±10 μm, (**b**) rotated about *X*-direction in the range of ±10 arcsec.

The last test was carried out to investigate the crosstalk errors between the outputs of the LMS when the detecting part was moved along the *X*- and *Y*-directions, and rotated about the *X*-, *Y*- and *Z*-directions, respectively. In order to avoid the influence of the crosstalk errors of the manual stages, a short movement range of about ±10 μm was applied to linear error and ±10 arcsec was applied to rotational error. Figure 10a shows the outputs of the LMS when the detecting part was moved along the *X*-direction. Variations of *δ_y_*, *θ_x_*, *θ_y,_* and *θ_z_*, which were approximately 0.8 μm, 2.2 arcsec, 0.5 arcsec and 1.8 arcsec, respectively, indicated the crosstalk errors of the LMS with respect to the *X*-directional motion of 20.0 μm. Similarly, variations were detected in the output of *δ_x_*, *δ_y_*, *θ_y,_* and *θ_z_* when an angular displacement *θ_x_* of 20.0 arcsec was applied to the detecting part with respect to the sensor head, as shown in Figure 10b. For the sake of clarity, the crosstalk errors of the LMS are summarized in Table 1. The crosstalk errors were quite large compared with the theoretical measurement resolutions of the LMS.

**Figure 11:**
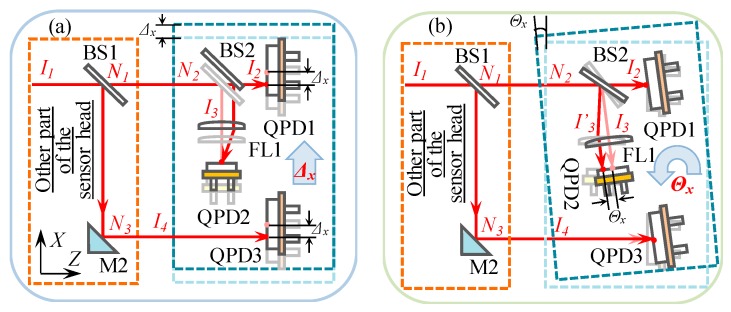
Optical path of the laser beam when the detecting part was applied to: (**a**) A linear displacement of Δ*_x_*, (**b**) an angular displacement of Θ*_x_*.

Confirmation of the reasons for generating the crosstalk errors was firstly carried out by analyzing the optical design of the LMS using the ray-tracing method, as shown in Figure 11. The laser beam *I*_1_ travels in the direction of the Z-axis with a direction vector of $I_{1}={\left[\begin{array}{ccc} 0 & 0 & 1 \end{array}\right]}^{T}$. The direction vector of reflection planes of BS1, BS2, and M2 can be expressed by:

(8) $$\begin{array}{l} N_{1}=N_{2}=\left[\begin{array}{ccc} N_{1x} & N_{1y} & N_{1z} \end{array}\right]={\left[\begin{array}{ccc} -\frac{\sqrt{2}}{2} & 0 & -\frac{\sqrt{2}}{2} \end{array}\right]}^{T}\\ N_{3}=\left[\begin{array}{ccc} N_{3x} & N_{3y} & N_{3z} \end{array}\right]={\left[\begin{array}{ccc} \frac{\sqrt{2}}{2} & 0 & \frac{\sqrt{2}}{2} \end{array}\right]}^{T} \end{array}$$

The transformation matrix of the reflection planes of the BS1, BS2, and M2 can be obtained as follows:

(9) $$B_{1}=B_{2}=B_{3}=\left[\begin{array}{ccc} 1-2N_{ix}^{2} & -2N_{ix}N_{iy} & -2N_{ix}N_{iz}\\ -2N_{ix}N_{iy} & 1-2N_{iy}^{2} & -2N_{iy}N_{iz}\\ -2N_{ix}N_{iz} & -2N_{iy}N_{iz} & 1-2N_{iz}^{2} \end{array}\right]\;=\left[\begin{array}{ccc} 0 & 0 & -1\\ 0 & 1 & 0\\ -1 & 0 & 0 \end{array}\right].$$

Thus, the direction vectors *I*_2_, *I*_3_, and *I*_4_ can be expressed as

(10) $$I_{2}=I_{1}={\left[\begin{array}{ccc} 0 & 0 & 1 \end{array}\right]}^{T},$$

(11) $$I_{3}=B_{2}\cdot I_{1}={\left[\begin{array}{ccc} -1 & 0 & 0 \end{array}\right]}^{T},$$

(12) $$I_{4}=B_{1}\cdot B_{3}\cdot I_{1}={\left[\begin{array}{ccc} 0 & 0 & 1 \end{array}\right]}^{T}.$$

When the detecting part of the LMS was applied to a linear displacement of Δ*_x_*, shown in Figure 11a, the direction vectors *I*_2_, *I_3,_* and *I*_4_ did not vary. Therefore, it was verified that the output of linear errors will not affect that of the angular errors in the designed LMS.

An angular displacement of Θ*_x_* was, then, applied to the detecting part, as shown in Figure 11b. The direction vector *N*_3_ of the BS2’s reflection plane was influenced by the angular displacement. *N*_3_ will be varied to be

(13) $$\;N_{3}^{\prime }=\left[\begin{array}{ccc} N_{3x}^{\prime } & N_{3y}^{\prime } & N_{3z}^{\prime } \end{array}\right]\;={\left[\begin{array}{ccc} \frac{\sqrt{2}}{2}(1-\Theta_{x}) & 0 & -\frac{\sqrt{2}}{2}(1+\Theta_{x}) \end{array}\right]}^{T}.$$

Thus, the effect of Θ*_x_* on *I*_3_ can be evaluated to be

(14) $$I_{3}^{R}=B_{2}^{R}\cdot I_{1}=\left[\begin{array}{ccc} 1-2N\prime_{3x}^{2} & -2N\prime_{3x}N\prime_{3y} & -2N\prime_{3x}N\prime_{3z}\\ -2N\prime_{3x}N\prime_{3y} & 1-2N\prime_{3y}^{2} & -2N\prime_{3y}N\prime_{3z}\\ -2N\prime_{3x}N\prime_{3z} & -2N\prime_{3y}N\prime_{3z} & 1-2N\prime_{3z}^{2} \end{array}\right]\cdot \left[\begin{array}{c} 0\\ 0\\ 1 \end{array}\right]\;=\left[\begin{array}{c} -1\\ 0\\ \Theta_{x} \end{array}\right].$$

Θ*_x_* can be detected by AC1 based on Equation (3). However, the direction vectors *I*_2_ and *I*_4_ were not changed by the angular displacement, which meant that the outputs of angular errors would also not affect those of the linear errors in the designed LMS.

The main error sources that caused the crosstalk errors were deemed the systematic errors, including the manufacture errors and installation errors of the optical components. In order to eliminate the influence of the systematic errors on the crosstalk errors, a compensation model, referred to as the modified measurement model, was proposed as follows,

(15) $$\left[\begin{array}{l} \delta_{x-L}\\ \delta_{y-L}\\ \theta_{x-L}\\ \theta_{y-L}\\ \theta_{z-L} \end{array}\right]=\left[\begin{array}{lllll} A_{1} & A_{2} & A_{3} & A_{4} & A_{5}\\ B_{1} & B_{2} & B_{3} & B_{4} & B_{5}\\ C_{1} & C_{2} & C_{3} & C_{4} & C_{5}\\ D_{1} & D_{2} & D_{3} & D_{4} & D_{5}\\ E_{1} & E_{2} & E_{3} & E_{4} & E_{5} \end{array}\right]\cdot \left[\begin{array}{l} \delta \prime_{x}\\ \delta \prime_{y}\\ \theta \prime_{x}\\ \theta \prime_{y}\\ \theta \prime_{z} \end{array}\right]=F\cdot \left[\begin{array}{l} \delta \prime_{x}\\ \delta \prime_{y}\\ \theta \prime_{x}\\ \theta \prime_{y}\\ \theta \prime_{z} \end{array}\right]$$

where, *M_n_* (*M* = *A*, *B*, *C*, *D* and *E*, *n* = 1, 2, 3, 4, and 5) are the coefficients of the crosstalk errors. *δ_x-L_*, *δ_y-L_*, *θ_x-L_*, *θ_y-L_* and *θ_z-L_* represent the actual five-DOF error motions of the linear stage, respectively. *δ′_x_*, *δ′_y_*, *θ′_x_*, *θ′_y_* and *θ′_z_* are the outputs of the LMS with the compensations of the detector sensitivity and dual-beam non-parallelism based on Equations (2) and (6). According to experimental results shown in Figure 10, the error coefficients of the designed LMS can be estimated to be

(16) $$F=\left[\begin{array}{ccccc} 1 & 0.018 & -0.108 & 0.021 & 0.016\\ -0.078 & 1 & 0.127 & -0.142 & -0.007\\ 0.126 & -0.010 & 1 & -0.149 & -0.105\\ -0.008 & 0.048 & -0.081 & 1 & 0.036\\ 0.084 & 0.277 & -0.038 & -0.122 & 1 \end{array}\right].$$

Table 2 shows the crosstalk errors of the LMS after compensation based on Equations (15) and (16). It can be seen that the crosstalk errors were significantly removed from the outputs of the LMS. The residual errors shown in Table 2 were dominated by the influence of the stability of the designed measurement system and the electronic noise of the photoelectric processing circuit.

## 4. Measurement of Error Motions of a Linear Stage

The performance investigations of the designed LMS for measuring the error motions of a linear stage were carried out. The travel distance of the linear stage is 1.2 m. The moving stage of the linear stage was manually moved approximately 120 mm in steps to 1.08 m. It should be noted that the crosstalk errors in the following experimental results were compensated by using the modified measurement model.

**Figure 12:**
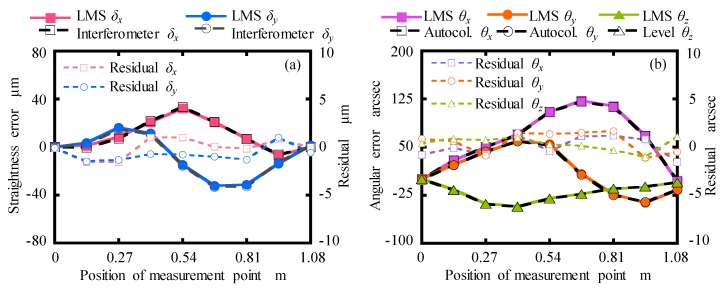
Error motions of the linear stage: (**a**) *δ_x_* and *δ_y_*, (**b**) *θ_x_*, *θ_y_*, and *θ_z_*.

The measurement experiments of the straightness errors, pitch and yaw errors and roll error were conducted by the comparison between the constructed LMS and high-precision commercial measurement instruments, including a laser interferometer (Agilent 5530, Keysight Technologies, Santa Clara, CA, USA) for measuring the straightness errors, the autocollimator for measuring the pitch and yaw errors, and the electrical level for measuring the roll error. The measured results are shown in Figure 12. It can be seen that the measured results by using the designed LMS were in a good agreement with the detected results of the commercial instruments. It has also been confirmed that the error motions measurements have good repeatability by five measurements although the figures are omitted for the sake of brevity. The maximum residuals for five measurements were calculated to be within ±1.5 μm, ±1.5 μm, ±1.8 arcsec, ±1.7 arcsec and ±1.5 arcsec corresponding to *δ_x_*, *δ_y_*, *θ_y_*, *θ_y,_* and *θ_z_*, respectively.

The experimental results show good measurement accuracy and repeatability of the designed LMS. It was confirmed that the designed LMS and modified measurement model have the potential to simultaneously measure the five-DOF error motions of a linear stage in a non-environmental controlled open laboratory.

## 5. Conclusions

This paper presented a newly designed laser measurement system (LMS), consisting of a sensor head and a detecting part, for simultaneously measuring five-DOF error motions (horizontal and vertical straightness errors, pitch error, yaw error and roll error) of linear stages. The measurement principles were described. The measurement errors of the LMS, which were induced by the variation of detector sensitivity, the laser beam drifted angular errors, the two-laser beam non-parallelism and the crosstalk errors, have been analyzed in detail, from which the measurement model was modified. A prototype of the LMS was constructed, in which a low-cost laser diode was used as the laser source. The effectiveness of the designed LMS and the modified measurement model were verified by a series of calibration and comparison experiments. Based on the comparison experiments of measuring the error motion of a linear stage, the residuals of the straightness errors, pitch error, yaw error and roll error were less than ±1.5 μm, ±1.8 arcsec, ±1.7 arcsec and ±1.5 arcsec compared with the laser interferometer, autocollimator and electrical level. The improvement of measurement accuracy and the uncertainty analysis will be carried out in the future research.

## Figures and Tables

**Table 1:** Crosstalk errors of the LMS without compensation.

	*δ_x_* [μm]	*δ_y_* [μm]	*θ_x_* [arcsec]	*θ_y_* [arcsec]	*θ_z_* [arcsec]
Moved → *X*-direction	**20.0**	0.8	2.2	0.5	1.8
Moved → *Y*-direction	2.1	**20.0**	2.6	3.0	0.5
Rotated → *X*-axis	2.1	0.5	**20.0**	2.6	2.7
Rotated → *Y*-axis	0.3	1.1	1.8	**20.0**	0.8
Rotated → *Z*-axis	1.4	5.2	0.7	2.8	**20.0**

**Table 2:** Crosstalk errors of the LMS with compensation.

	*δ_x_* [μm]	*δ_y_* [μm]	*θ_x_* [arcsec]	*θ_y_* [arcsec]	*θ_z_* [arcsec]
Moved → *X*-direction	**20.0**	0.5	0.3	0.3	0.8
Moved → *Y*-direction	0.8	**20.0**	0.5	0.3	0.5
Rotated → *X*-axis	0.3	0.2	**20.0**	0.6	0.8
Rotated → *Y*-axis	0.3	0.2	0.2	**20.0**	0.1
Rotated → *Z*-axis	0.6	6.1	0.3	0.9	**20.0**
